# Supplementary material for: Building heat-resilient Caribbean reefs: integrating thermal thresholds and coral colonies selection in restoration
Source: PeerJ. 2025 Sep 5;13:e19987. doi: 10.7717/peerj.19987 (PMC12422269; doi:10.7717/peerj.19987)

**Appendix S1**

**Figure S1:** Collection sites in the southeastern region of the Dominican Republic: Coco Reef: 18.33668N, 68.8239W; Playita: 18.3728N, 68.8532W; and Magayan: 18.3609N, 68.8453W.

**
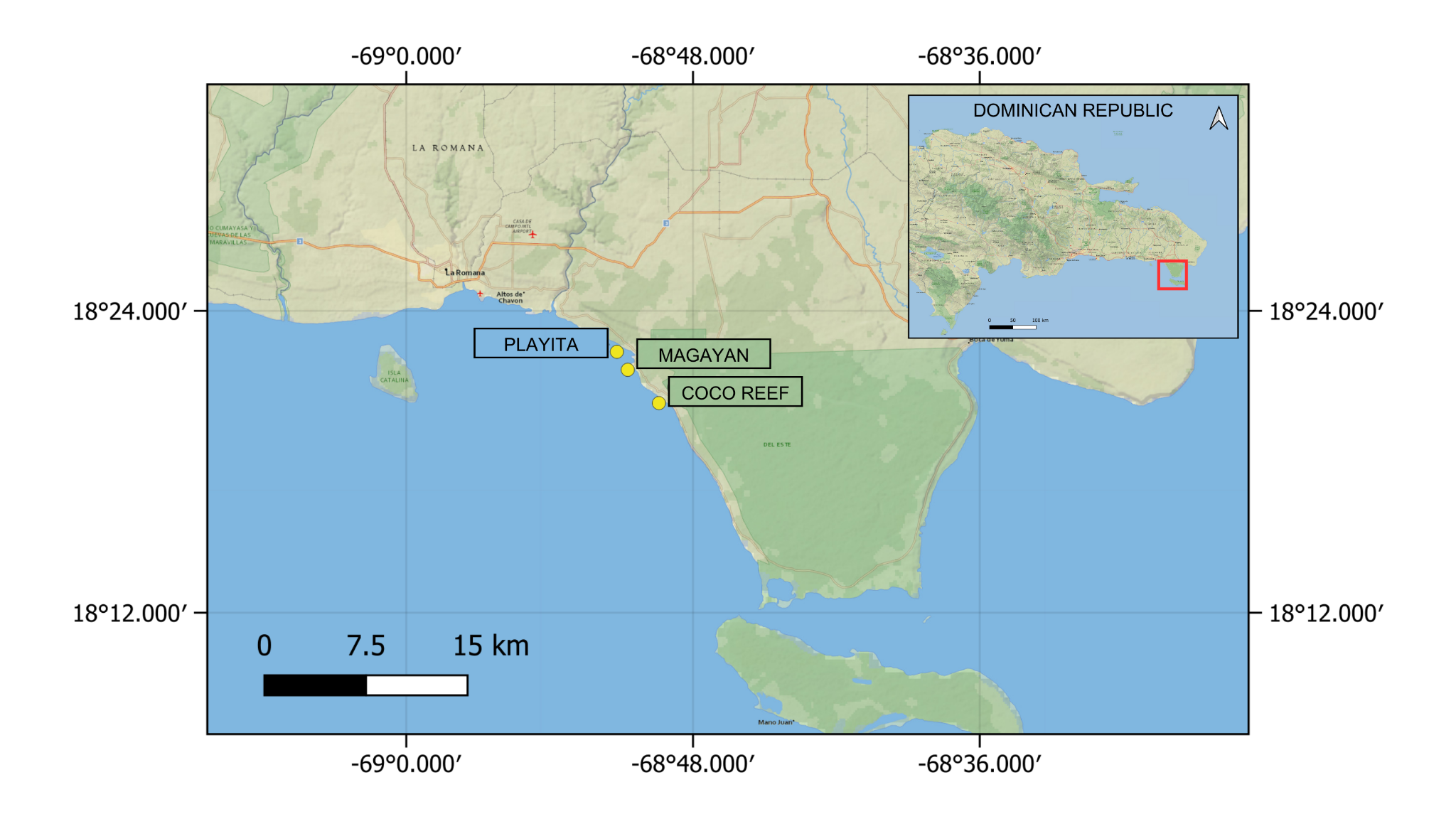
**

**Table S1:** Experiment dates for each experimental phase and species. ACER: *Acropora cervicornis*; DLAB: *Diploria labyrinthiformis*; MCAV: *Montastraea cavernosa*; OANN: *Orbicella annularis*; OFAV: *Orbicella faveolata*; PAST: *Porites astreoides*; PPOR: *Porites porites*.

| **Experiment phase** | **species** | **Experiment month** | **Experiment year** |
| --- | --- | --- | --- |
| I: interspecific | ACER | March | 2021 |
|  | DLAB | April | 2021 |
|  | OFAV | April | 2021 |
|  | OANN | May | 2021 |
|  | MCAV | May | 2021 |
|  | PPOR | May | 2021 |
|  | PAST | October | 2021 |
| II: intraspecific | DLAB | November | 2021 |
|  | OFAV | November | 2021 |
|  | OANN | February | 2022 |
|  | PAST | March | 2022 |
|  | MCAV | June | 2022 |

**Table S2:** Results of Nemenyi post-hoc test for photosynthetic efficiency between species across all temperatures.

**ACER DLAB MCAV OANN OFAV PAST**

**DLAB 0.00016 - - - - -**

**MCAV 1.5e-06 0.97088 - - - -**

**OANN 6.3e-08 0.77017 0.99813 - - -**

**OFAV 5.6e-07 0.93234 1.00000 0.99980 - -**

**PAST 4.4e-08 0.65910 0.98947 0.99999 0.99742 -**

**PPOR 1.00000 0.00015 1.4e-06 5.9e-08 5.3e-07 4.2e-08**

**Table S3:** Interspecific data analysis - Kruskall-Wallis Chi-square values (**𝛘^2^**) and significance (*p*-value), and significant pairs (*p*<0.05) obtained with Nemenyi tests - differences in photosynthetic efficiency (*F_v_/F_m_*) and pixel intensity between species for each temperature treatment.

|  | **FvFm** | | | **Pixel intensity** | | |
| --- | --- | --- | --- | --- | --- | --- |
| **Temperature** | **𝛘^2^**  **(df = 6)** | ***p*-value** | **Significant pairs** | **𝛘^2^**  **(df = 6)** | ***p*-value** | **Significant pairs** |
| MMM (28.5°C) | 24.529 | 0.0004172 | ACER - MCAV  ACER - PAST  MCAV - PPOR | 41.529 | 2.277e-07 | ACER - DLAB  ACER - MCAV  ACER - OANN  ACER - OFAV  ACER - PAST |
| MMM+3 (31.5°C) | 27.447 | 0.0001194 | ACER - MCAV  ACER - OANN  ACER - PAST  PAST - PPOR | 44.115 | 7.015e-08 | ACER - DLAB  ACER - MCAV  ACER - OANN  ACER - OFAV  ACER - PAST |
| MMM+4 (32.5°C) | 17.894 | 0.006504 | ACER - OANN  OANN - PPOR | 39.587 | 5.491e-07 | ACER - DLAB  ACER - MCAV  ACER - OANN  ACER - PAST |
| MMM+5 (33.5°C) | 31.953 | 1.666e-05 | ACER - OANN  ACER - OFAV  ACER - DLAB  OANN - PPOR  ACER - PAST  PAST - PPOR | 45.545 | 3.647e-08 | ACER - MCAV  ACER - OANN  ACER - PAST  ACER - PPOR  DLAB - PPOR  OFAV - PPOR |
| MMM+6 (34.5°C) | 30.112 | 3.742e-05 | ACER - MCAV  ACER - OFAV  ACER - PAST  PPOR - MCAV  PPOR - OFAV  PPOR - PAST | 51.356 | 2.513e-09 | ACER - MCAV  ACER - OANN  ACER - OFAV  ACER - PAST  ACER - PPOR  DLAB - OANN  DLAB - PPOR |
| MMM+7 (35.5°C) | 42.239 | 1.649e-07 | ACER - MCAV  ACER - OFAV  ACER - PAST  ACER - OANN  DLAB - OANN  PPOR - OANN  PPOR - OFAV  PPOR - PAST | 44.484 | 5.926e-08 | ACER - MCAV  ACER - OANN  ACER - OFAV  ACER - PAST  ACER - PPOR  DLAB - PPOR |
| MMM+8 (36.5°C) | 46.468 | 2.388e-08 | ACER - OANN  ACER - OFAV  MCAV - OANN  MCAV - OFAV  OANN - PAST  PPOR - DLAB  PPOR - OANN  PPOR - OFAV | 29.154 | 5.688e-05 | ACER - MCAV  ACER - OANN  ACER - OFAV  ACER - PAST  ACER - PPOR |
| MMM+9 (37.5°C) | 35.659 | 3.211e-06 | ACER - DLAB  ACER - MCAV  ACER - OANN  ACER - OFAV  PPOR - MCAV  PPOR - OANN  PPOR - OFAV | 36.262 | 2.451e-06 | ACER - DLAB  ACER - MCAV  ACER - OANN  ACER - OFAV  ACER - PPOR  OFAV - PAST |

**Table S4:** Photosynthetic efficiency (*F_v_/F_m_*) averaged values by temperature and median values.

| **species** | **temperature (°C)** | **temperature code** | **FvFm** | **median** |
| --- | --- | --- | --- | --- |
| ACER | 28.5 | MMM | 0.59 | 0.58 |
|  | 31.5 | MMM+3 | 0.62 |  |
|  | 32.5 | MMM+4 | 0.59 |  |
|  | 33.5 | MMM+5 | 0.60 |  |
|  | 34.5 | MMM+6 | 0.58 |  |
|  | 35.5 | MMM+7 | 0.55 |  |
|  | 36.5 | MMM+8 | 0.42 |  |
|  | 37.5 | MMM+9 | 0.44 |  |
| DLAB | 28.5 | MMM | 0.57 | 0.52 |
|  | 31.5 | MMM+3 | 0.57 |  |
|  | 32.5 | MMM+4 | 0.54 |  |
|  | 33.5 | MMM+5 | 0.53 |  |
|  | 34.5 | MMM+6 | 0.51 |  |
|  | 35.5 | MMM+7 | 0.45 |  |
|  | 36.5 | MMM+8 | 0.26 |  |
|  | 37.5 | MMM+9 | 0.19 |  |
| MCAV | 28.5 | MMM | 0.51 | 0.48 |
|  | 31.5 | MMM+3 | 0.55 |  |
|  | 32.5 | MMM+4 | 0.54 |  |
|  | 33.5 | MMM+5 | 0.52 |  |
|  | 34.5 | MMM+6 | 0.45 |  |
|  | 35.5 | MMM+7 | 0.39 |  |
|  | 36.5 | MMM+8 | 0.45 |  |
|  | 37.5 | MMM+9 | 0.14 |  |
| OANN | 28.5 | MMM | 0.57 | 0.53 |
|  | 31.5 | MMM+3 | 0.56 |  |
|  | 32.5 | MMM+4 | 0.53 |  |
|  | 33.5 | MMM+5 | 0.52 |  |
|  | 34.5 | MMM+6 | 0.55 |  |
|  | 35.5 | MMM+7 | 0.25 |  |
|  | 36.5 | MMM+8 | 0.15 |  |
|  | 37.5 | MMM+9 | 0.15 |  |
| OFAV | 28.5 | MMM | 0.57 | 0.52 |
|  | 31.5 | MMM+3 | 0.58 |  |
|  | 32.5 | MMM+4 | 0.55 |  |
|  | 33.5 | MMM+5 | 0.54 |  |
|  | 34.5 | MMM+6 | 0.51 |  |
|  | 35.5 | MMM+7 | 0.30 |  |
|  | 36.5 | MMM+8 | 0.20 |  |
|  | 37.5 | MMM+9 | 0.16 |  |
| PAST | 28.5 | MMM | 0.53 | 0.49 |
|  | 31.5 | MMM+3 | 0.52 |  |
|  | 32.5 | MMM+4 | 0.54 |  |
|  | 33.5 | MMM+5 | 0.48 |  |
|  | 34.5 | MMM+6 | 0.46 |  |
|  | 35.5 | MMM+7 | 0.33 |  |
|  | 36.5 | MMM+8 | 0.37 |  |
|  | 37.5 | MMM+9 | 0.20 |  |
| PPOR | 28.5 | MMM | 0.60 | 0.59 |
|  | 31.5 | MMM+3 | 0.60 |  |
|  | 32.5 | MMM+4 | 0.60 |  |
|  | 33.5 | MMM+5 | 0.59 |  |
|  | 34.5 | MMM+6 | 0.58 |  |
|  | 35.5 | MMM+7 | 0.52 |  |
|  | 36.5 | MMM+8 | 0.52 |  |
|  | 37.5 | MMM+9 | 0.40 |  |

**Table S5:** Results of Nemenyi post-hoc test for pixel intensity between species across all temperatures.

**ACER DLAB MCAV OANN OFAV PAST**

**DLAB 5.8e-14 - - - - -**

**MCAV < 2e-16 0.0439 - - - -**

**OANN < 2e-16 0.0036 0.9901 - - -**

**OFAV 7.4e-14 0.9302 0.4808 0.1146 - -**

**PAST 5.4e-14 0.9219 0.5534 0.1549 1.0000 -**

**PPOR 6.7e-14 0.2954 0.9857 0.7253 0.9265 0.9496**

**Table S6:** Pixel intensity averaged values by temperature and median values.

| **species** | **temperature (°C)** | **temperature code** | **pixel intensity** | **median** |
| --- | --- | --- | --- | --- |
| ACER | 28.5 | MMM | 168.80 | 190.27 |
|  | 31.5 | MMM+3 | 166.70 |  |
|  | 32.5 | MMM+4 | 173.74 |  |
|  | 33.5 | MMM+5 | 187.88 |  |
|  | 34.5 | MMM+6 | 192.66 |  |
|  | 35.5 | MMM+7 | 247.06 |  |
|  | 36.5 | MMM+8 | 254.82 |  |
|  | 37.5 | MMM+9 | 253.48 |  |
| DLAB | 28.5 | MMM | 80.09 | 104.45 |
|  | 31.5 | MMM+3 | 78.08 |  |
|  | 32.5 | MMM+4 | 75.29 |  |
|  | 33.5 | MMM+5 | 81.32 |  |
|  | 34.5 | MMM+6 | 127.59 |  |
|  | 35.5 | MMM+7 | 131.88 |  |
|  | 36.5 | MMM+8 | 137.14 |  |
|  | 37.5 | MMM+9 | 136.06 |  |
| MCAV | 28.5 | MMM | 60.69 | 80.59 |
|  | 31.5 | MMM+3 | 70.04 |  |
|  | 32.5 | MMM+4 | 68.55 |  |
|  | 33.5 | MMM+5 | 64.83 |  |
|  | 34.5 | MMM+6 | 91.14 |  |
|  | 35.5 | MMM+7 | 101.53 |  |
|  | 36.5 | MMM+8 | 109.18 |  |
|  | 37.5 | MMM+9 | 121.55 |  |
| OANN | 28.5 | MMM | 60.95 | 68.09 |
|  | 31.5 | MMM+3 | 61.80 |  |
|  | 32.5 | MMM+4 | 66.94 |  |
|  | 33.5 | MMM+5 | 65.69 |  |
|  | 34.5 | MMM+6 | 69.24 |  |
|  | 35.5 | MMM+7 | 100.32 |  |
|  | 36.5 | MMM+8 | 122.04 |  |
|  | 37.5 | MMM+9 | 126.22 |  |
| OFAV | 28.5 | MMM | 81.40 | 86.41 |
|  | 31.5 | MMM+3 | 81.93 |  |
|  | 32.5 | MMM+4 | 83.16 |  |
|  | 33.5 | MMM+5 | 81.68 |  |
|  | 34.5 | MMM+6 | 89.66 |  |
|  | 35.5 | MMM+7 | 101.39 |  |
|  | 36.5 | MMM+8 | 113.76 |  |
|  | 37.5 | MMM+9 | 111.81 |  |
| PAST | 28.5 | MMM | 78.66 | 88.36 |
|  | 31.5 | MMM+3 | 70.73 |  |
|  | 32.5 | MMM+4 | 71.04 |  |
|  | 33.5 | MMM+5 | 87.49 |  |
|  | 34.5 | MMM+6 | 98.75 |  |
|  | 35.5 | MMM+7 | 112.41 |  |
|  | 36.5 | MMM+8 | 153.03 |  |
|  | 37.5 | MMM+9 | 222.44 |  |
| PPOR | 28.5 | MMM | 92.85 | 93.84 |
|  | 31.5 | MMM+3 | 96.84 |  |
|  | 32.5 | MMM+4 | 94.84 |  |
|  | 33.5 | MMM+5 | 52.57 |  |
|  | 34.5 | MMM+6 | 58.73 |  |
|  | 35.5 | MMM+7 | 72.04 |  |
|  | 36.5 | MMM+8 | 111.79 |  |
|  | 37.5 | MMM+9 | 144.49 |  |

**Figure S2:** Biplot of Principal Component Analysis (PCA) of all colonies across five coral species based on three heat stress metrics: photosynthetic efficiency (Fv/Fm), red channel pixel intensity, and visual bleaching scores. Values for pixel intensity and bleaching scores were directionally inverted to ensure that higher values represent greater thermotolerance across all metrics. PC1 explains the majority of the variance, primarily driven by visual bleaching and pixel intensity, while Fv/Fm contributes less.

**
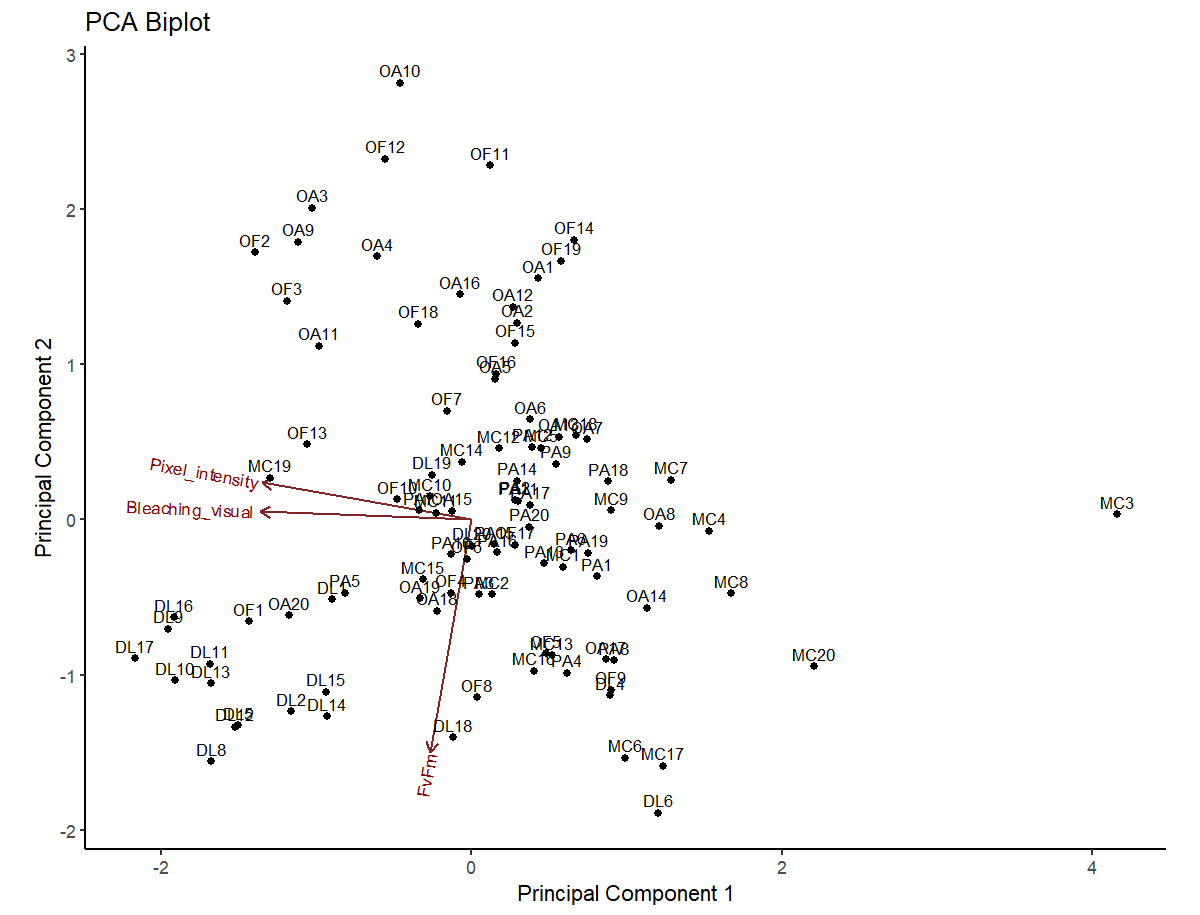
**

**Figure S3:** Biplot of Principal Component Analysis (PCA) of all colonies across five coral species based on three heat stress metrics: photosynthetic efficiency (Fv/Fm), red channel pixel intensity, and visual bleaching scores. Colonies are grouped and color-coded by species. Although species-level clustering is not fully discrete, there is visible grouping, suggesting that species identity may influence the multivariate structure of the thermotolerance metrics.


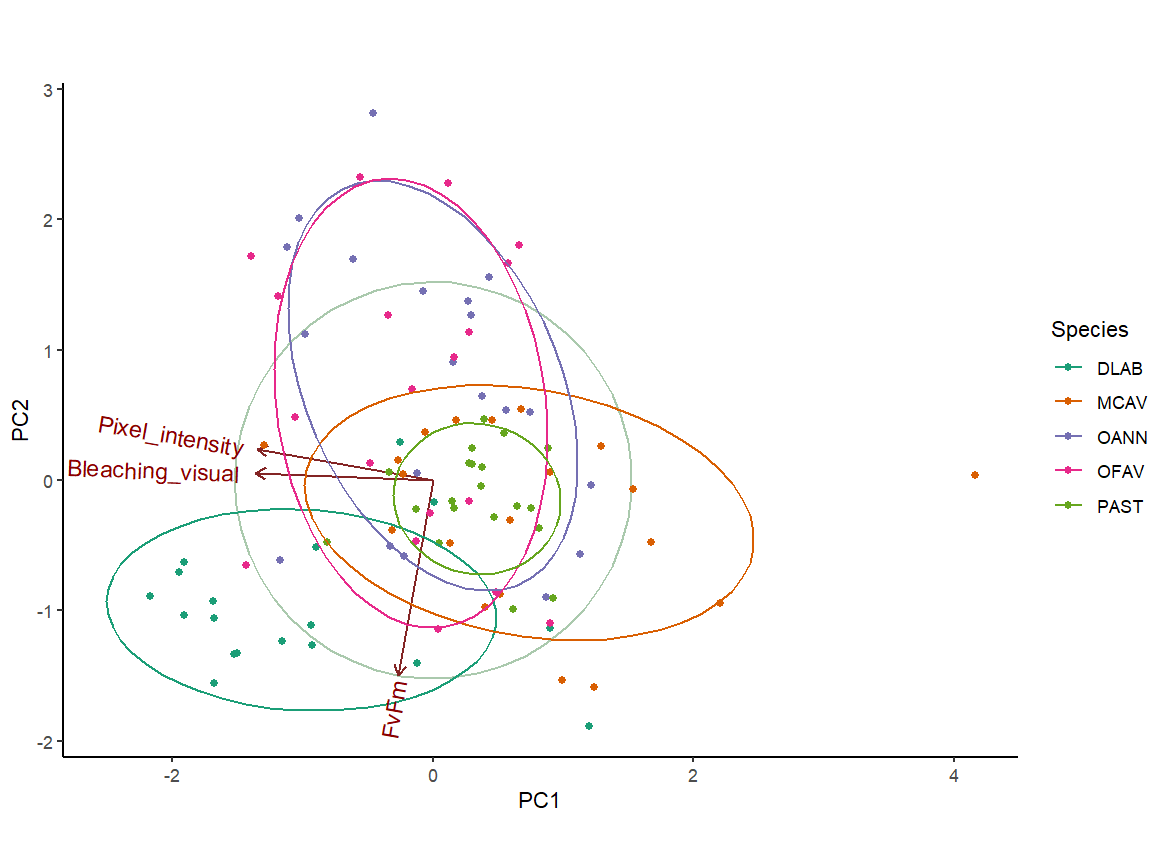


**Table S7:** Results of Kruskal-Wallis tests evaluating differences in thermotolerance scores among source reefs for each species. Chi-square values (**𝛘^2^**) and significance (*p*-value).

| **Species** | **𝛘^2^**  **(df )** | ***p*-value** |
| --- | --- | --- |
| DLAB | 0.046259 (2) | 0.9771 |
| MCAV | 2.9221 (2) | 0.232 |
| OANN | 0.28712 (1) | 0.5921 |
| OFAV | 4.6139 (2) | 0.09956 |
| PAST | 0.65525 (2) | 0.7206 |

**Figure S4:** Thermotolerance scores across species and reefs. COCO: Coco Reef; MAGA: Magayan; PLAY: Playita.


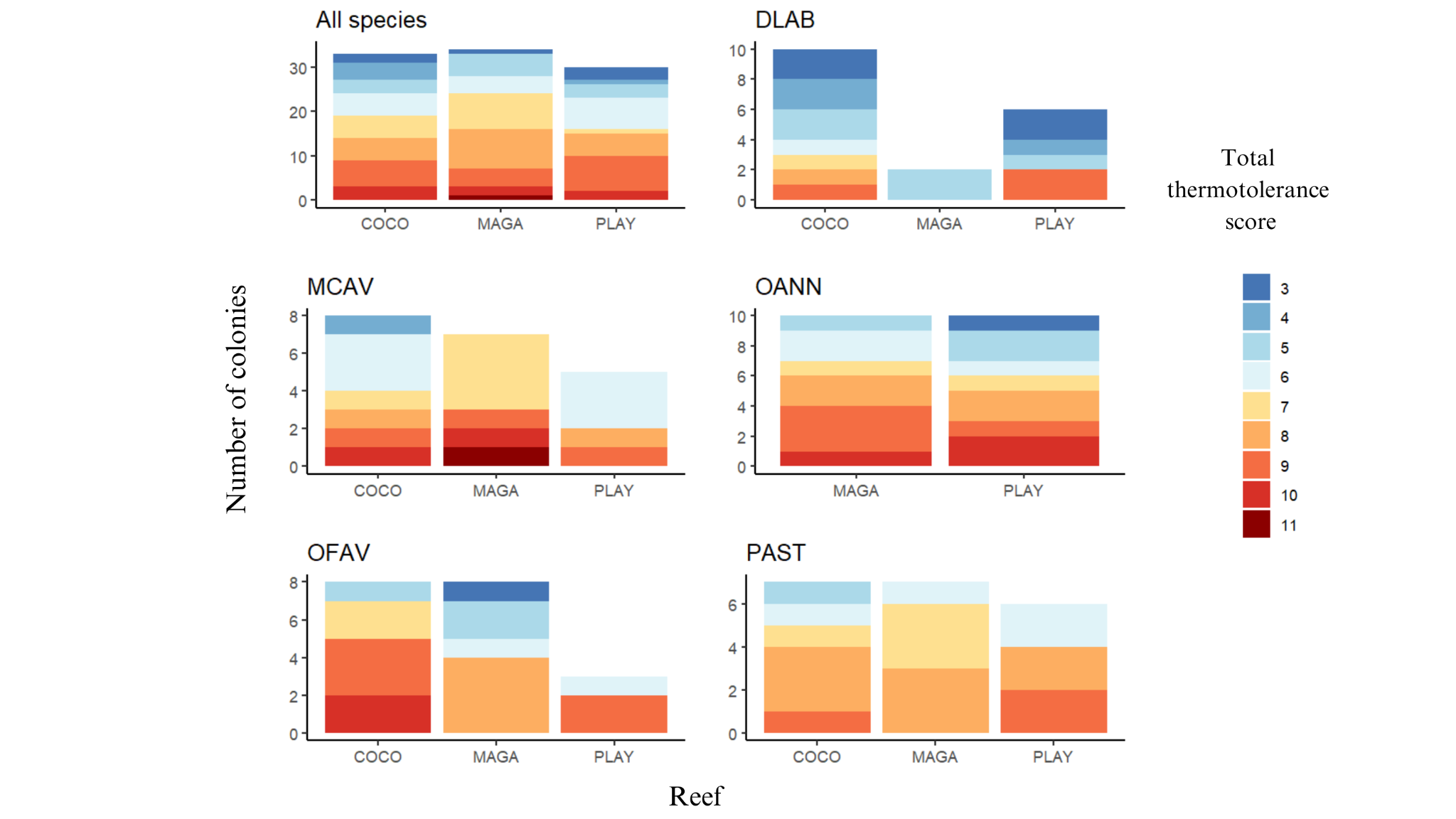

Supplement: Supplemental Information 1 — Sample collection dates and sites; backup statistics: Kruskal–Wallis tests and post-hoc results; averaged raw values and median of Fv/Fm and pixel intensity on which T 50 selection is based; Principal Component Analysis (PCA) of colonies across species and heat stress metrics; Thermotolerance scores across species and reefs. [file peerj-13-19987-s001.docx]
