# Supplementary material for: Building heat-resilient Caribbean reefs: integrating thermal thresholds and coral colonies selection in restoration
Source: PeerJ. 2025 Sep 5;13:e19987. doi: 10.7717/peerj.19987 (PMC12422269; doi:10.7717/peerj.19987)
Supplement: Supplemental Information 2 — Summary and step-by-step instructions for intraspecific thermotolerance scoring. [file peerj-13-19987-s002.docx]

**Appendix S2**

**Figure S1:** Summary of thermotolerance scoring procedure for each of the heat stress evaluation methods


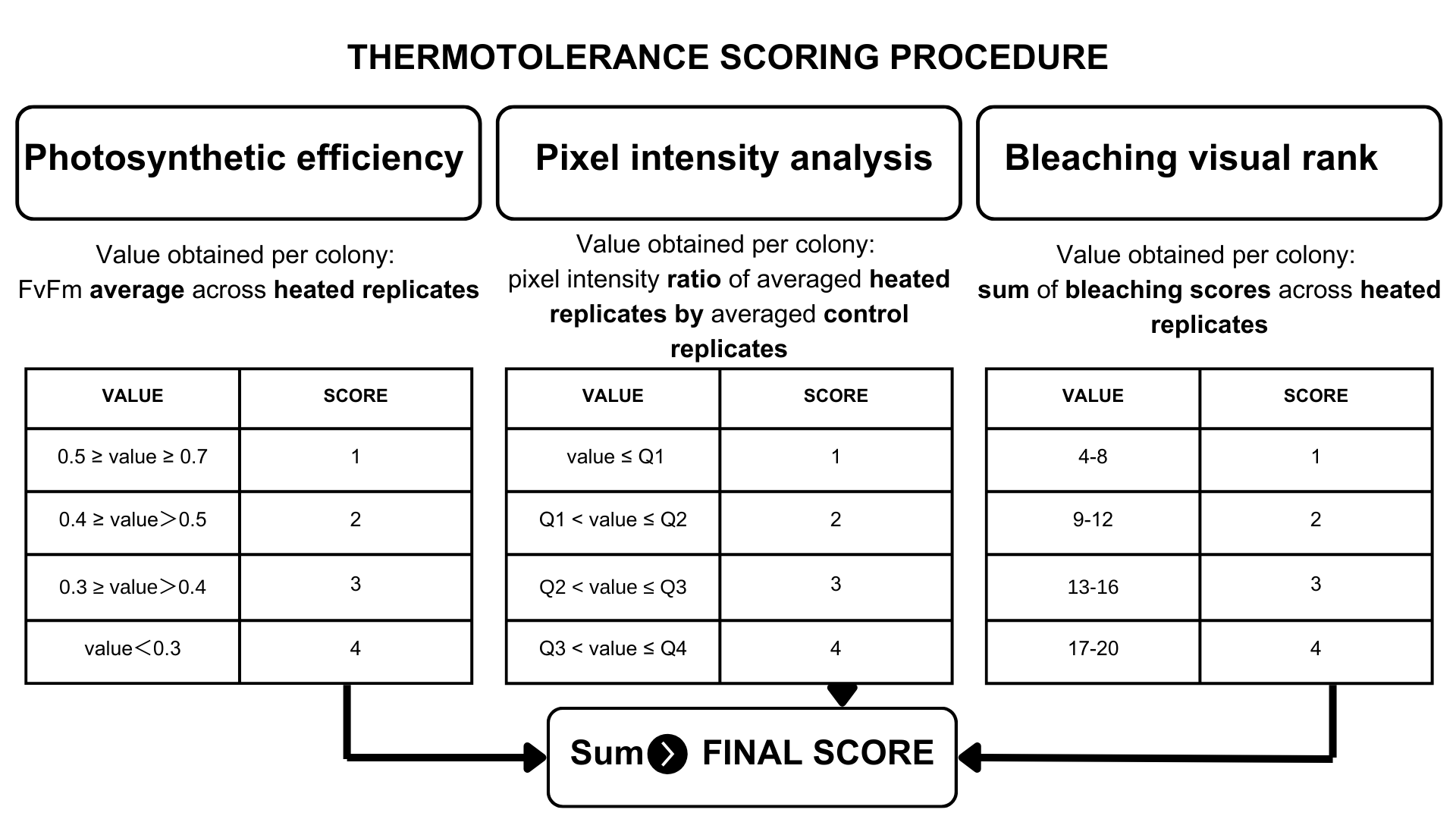


**Figure S2:** Visual bleaching scores with picture examples for each of the species of the study. Scoring was performed before the pictures were taken, as coloration differences were best appreciated when the samples were submerged in water. Example images were previously calibrated to balance white levels except for score 5 (totally bleached) in *Acropora cervicornis,* since in the calibrated image, the fragment was barely noticeable.


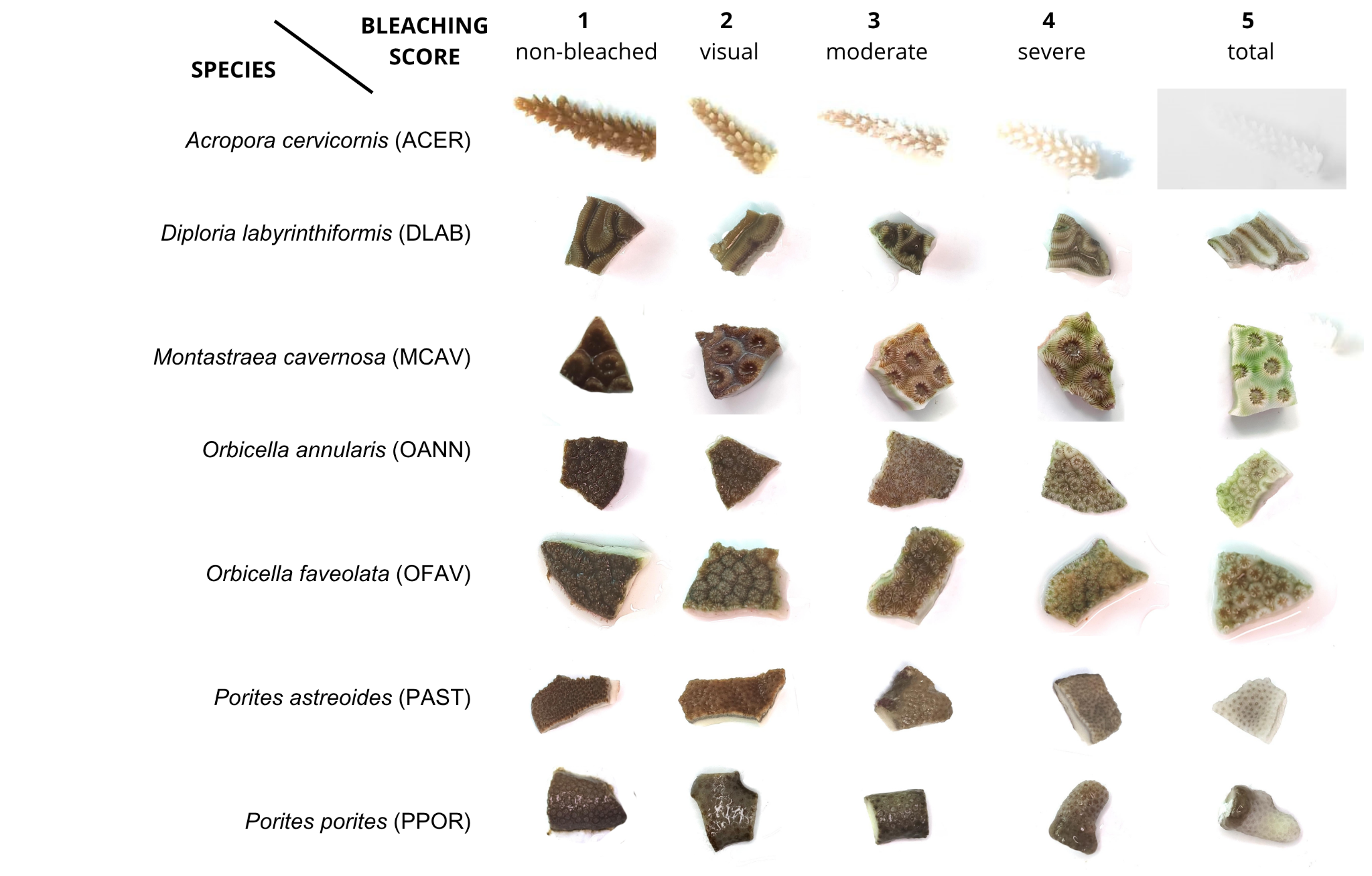


**THERMOTOLERANCE SCORES PROCEDURES**

**(PHASE II - intraspecific)**

There are three methods that will be used to estimate the heat stress per colony: bleaching visual ranking, pixel intensity analysis, and PAM fluorescence.

**A. Bleaching Visual Ranking .**

A rank from 1 (not bleached) to 5 (fully bleached) is assigned to each replicate the morning after the T50 experiments. Therefore, each colony will have 4 rank numbers, one per replicate.

1. Organize the Bleaching Visual sheet by **colony** and **treatment.**


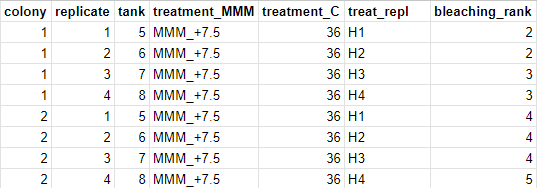


1. Add the 4 rank numbers for each replicate to get a **single sum number per colony**. For example, colony 1 has the following rank numbers for its replicates: 2, 2, 3, 3. When added, colony 1 would have a sum number of 10.
2. A **single thermotolerance score** per colony will be assigned using the sum number with the following table.

| **Sum number**  **(previously obtained by adding 4 replicates)** | **Thermotolerance score**  **to assign** |
| --- | --- |
| **4 - 8** | **1** |
| **9 - 12** | **2** |
| **13 - 16** | **3** |
| **17 - 20** | **4** |

To assign the thermotolerance score, the following IFS formula can copied and pasted in the visual_score column and used in the Bleaching Visual sheet:

*=IFS(I2<=8, 1, I2=9, 2, I2=10, 2, I2=11, 2, I2=12, 2, I2=13, 3, I2=14, 3, I2=15, 3, I2=16, 3, I2>=17, 4)**

** Make sure that the column used refers to the sum column. In this case it is column I.*

**The final result is a qualitative measurement of bleaching per colony, named the thermotolerance score. The smaller the score, the more thermotolerance.**

**B. Pixel Intensity Analysis .**

After the MATLAB image analysis, 8 red, 8 green and 8 blue pixel intensity measurements will be obtained per replicate, per colony. Therefore, colony 1 has 4 replicates, and each replicate has 8 measurements of pixel intensity. We will only use the red pixel intensities.

1. Organize the Image Analysis sheet by **colony**.
2. Obtain the average red pixel intensity per replicate, selecting the 8 measurements of pixel intensity (*=promedio(D2:D9)*). Each colony has 4 HEAT replicates, and should therefore have 4 average measurements.


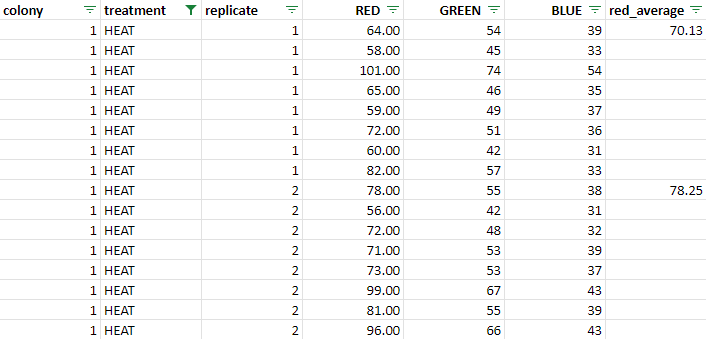


* Only showing 2 red_average measurements out of the total 4 for colony 1.

1. Copy and paste the **colony, treatment, replicate and red_average columns** into the ordered sheet. Remember to select paste values only when pasting the red_average column to keep the true numeric values. Remove extra spaces: select all data, click on Data, select Create filter. Use the filter in the red_average column to select all rows with spaces and delete these rows. Use filter to pick the rows with data.
2. Organize the Ordered sheet by **colony** and **treatment.**
3. Obtain a **new red average** (*=PROMEDIO(D2:D3)*) and **standard deviation** (*=DESVEST(D2:D3)*) per colony and treatment based on previous average measurements. Colony 1 should have a single average and standard deviation for its HEAT treatment and another for its CONTROL.


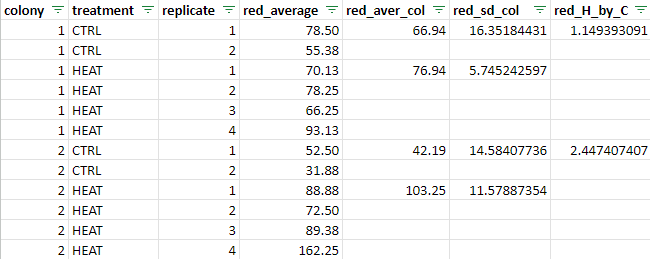


* Note that CONTROL only had two replicates per colony, while HEAT treatment had 4.

1. A **ratio** will be obtained per colony by **dividing** the red_aver_col measurement in the **HEAT treatment over the CONTROL treatment.** For example, for colony 1 this would be:

HEAT red_aver_col / CNTRL red_aver_col

76.94 / 66.94

This ratio is obtained because the original color in each colony varies. Some colonies are naturally lighter and some colonies are naturally darker. This variation can affect the red pixel intensity measurements after the HEAT experiments and needs to be taken into account through the ratio.

A single ratio value (red_H_by_C) will be obtained per colony.


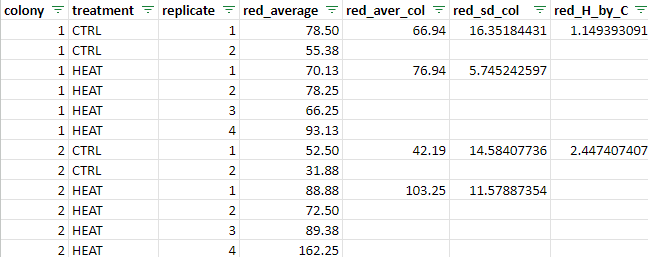


1. Copy and paste the **colony** and **red_H_by_C** columns into the Final Sheet. Use a filter to remove spaces from view in the red_H_by_C column.
2. Obtain the quartiles for the data using the red_H_by_C measurements: *=CUARTIL(B2:B29,1), =CUARTIL(B2:B29,2), =CUARTIL(B2:B29,3), =CUARTIL(B2:B29,4).* Each quartile can be calculated in a new column.
3. Using the Quartiles and the red_H_by_C measurements, manually assign each colony a **score** from 1 to 4. Use the following table:

| **If the ratio measurement**  **(red_H_by_C) is** | **Thermotolerance score**  **to assign** |
| --- | --- |
| Smaller than or equal to Q1  **X** < Q1 | **1** |
| Larger than Q1 - smaller than or equal to Q2  **X** > Q1 **OR** **X** ≤ Q2 | **2** |
| Larger than Q2 - smaller than or equal to Q3  **X** > Q2 **OR** **X** ≤ Q3 | **3** |
| Larger than Q3 - smaller or equal to Q4  **X** > Q3 **OR** **X** ≤ Q4 | **4** |

For example, the ratio for colony 1 is smaller than Q1. Therefore, score for colony 1 is 1. On the other hand, the ratio for colony 10 is equal to Q4 and consequently its score is a 4. The ratio for colony 8 is larger than Q2 and smaller than Q3; its score is a 3. These scores should be assigned in the thermal_score column.


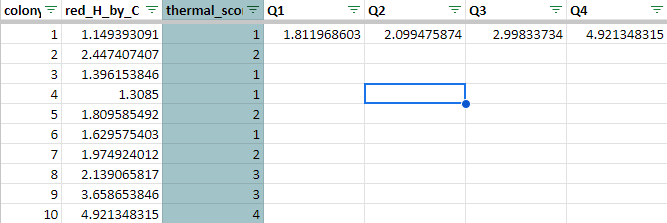


**The final result is a quantitative measurement of bleaching per colony, named the thermal score. The smaller the score, the more thermotolerance.**

**C. PAM Fluorescence .**

The photosynthetic efficiency of PSII, hereafter referred to as fv/fm, was obtained for each replicate and colony after the HEAT experiment. Therefore, each colony should have 4 fv/fm measurements.

1. Organize the Fluorometry sheet by **sample_ID (colony)** and **treatment.**
2. Obtain the **average fv/fm** (*=PROMEDIO(I3:I6)*) and **standard deviation** (*=DESVEST(I3:I6)*) **per colony** selecting the 4 measurements of each replicate. Each colony should have a single fv/fm average and standard deviation measurement (per treatment).


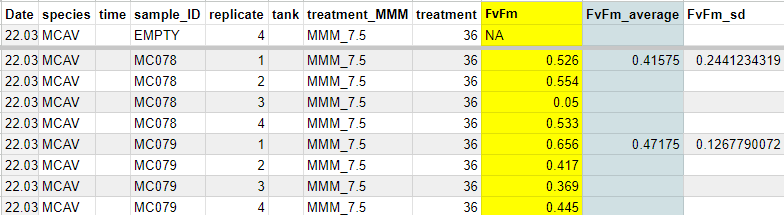


1. Assign the **tolerance score** manually per colony with the fv/fm average using the following table:

| **FvFm average** | **Thermotolerance score**  **to assign** |
| --- | --- |
| (including) 0.5 - 0.7 | **1** |
| (including) 0.4 - 0.5 (not including) | **2** |
| (including) 0.3 - 0.4 (not including) | **3** |
| < 0.3 | **4** |

* **ONLY TWO** decimals are considered.

For example, colony 1 (MC078) has an fv/fm average of 0.41575. Since only two decimals are considered, it rounds up to 0.42. Therefore, the score for colony 1 is 2. Colony 3 (MC080) has an fv/fm average of 0.308 that rounds up to 0.31. Therefore, its score is 3.


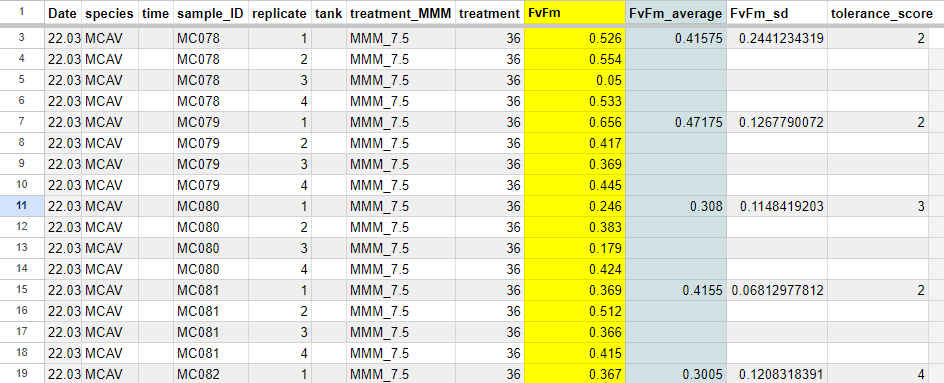


**The final result is a quantitative measurement of PSII efficiency per colony, which reflects stress, named the tolerance score. The smaller the score, the more thermotolerance.**

**FINAL RANKING TABLE**

A final ranking table with the scores across methods will be created to identify the species with the highest priority.

1. On a separate sheet, copy and paste the **reef, species, number of colony and colony ID columns** from the repository/main data sheet.
2. Copy and paste the **final scores** from each method (visual bleaching, red pixel intensity and fluorescence) for each colony.
3. Obtain the **Total Score** by adding the scores (*=SUMA(E2:G2)*) from each method for each colony.
4. Organize the table from the lowest to highest Total Score.
5. Assign the **priority** of each colony from 1 to 3: the first two numbers in total score receive a number 1 priority, the third number receives a number 2 priority, and the remaining numbers receive a number 3 priority.


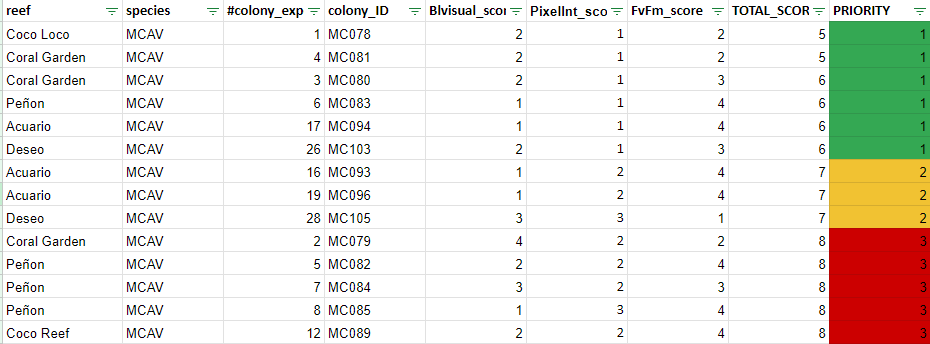


For example, the Total Score of colony 1 MC078 is 5, and was obtained by adding its Blvisual, Pixelint and FvFm scores. Once the Total Scores were organized, the first two Total Score numbers were 5 and 6. These received a number one priority score. The third Total Score number is 7, and received a number two priority score. All other Total Score numbers received a number three priority score.
